# Ionic Silver and Electrical Treatment for Susceptibility and Disinfection of *Escherichia coli* Biofilm-Contaminated Titanium Surface

**DOI:** 10.3390/molecules27010180

**Published:** 2021-12-28

**Authors:** Kritphudis Suttasattakrit, Arnon Khamkeaw, Chanchana Tangwongsan, Prasit Pavasant, Muenduen Phisalaphong

**Affiliations:** 1 Biomedical Engineering Program, Faculty of Engineering, Chulalongkorn University, Bangkok 10330, Thailand; kritphudiz@gmail.com; 2 Department of Chemical Engineering, Faculty of Engineering, Chulalongkorn University, Bangkok 10330, Thailand; arnon.k1231@gmail.com; 3 Department of Electrical Engineering, Faculty of Engineering, Chulalongkorn University, Bangkok 10330, Thailand; tchanchana@yahoo.com; 4 Center of Excellence for Regenerative Dentistry, Department of Anatomy, Faculty of Dentistry, Chulalongkorn University, Bangkok 10330, Thailand; prasit215@gmail.com

**Keywords:** biofilm, *E. coli*, titanium, disinfection, ionic silver, electrical treatment

## Abstract

In this work, surface disinfection and biofilm susceptibility were investigated by applying ionic silver of 0.4–1.6 µg/mL and cathodic voltage-controlled electrical treatment of 1.8 V and a current of 30 mA to *Escherichia coli* (*E. coli*) ATCC 25922 biofilm-contaminated titanium substrates. Herein, it is evident that the treatment exhibited the potential use to enhance the susceptibility of bacterial biofilms for surface disinfection. In vitro studies have demonstrated that the ionic silver treatment of 60 min significantly increased the logarithmic reduction (LR) of bacterial populations on disinfectant-treated substrates and the electrical treatment enhanced the silver susceptibility of *E. coli* biofilms. The LR values after the ionic silver treatments and the electric-enhanced silver treatments were in the ranges of 1.94–2.25 and 2.10–2.73, respectively. The treatment was also associated with morphological changes in silver-treated *E. coli* cells and biofilm-contaminated titanium surfaces. Nevertheless, the treatments showed no cytotoxic effects on the L929 mouse skin fibroblast cell line and only a slight decrease in pH was observed during the electrical polarization of titanium substrate.

## 1. Introduction

Biofilms are communities of bacteria that form when cells attach to a surface and secrete a matrix of extracellular polymeric substance (EPS). A hallmark characteristic of biofilms is their tolerance to antimicrobials. Killing microorganisms in biofilm can require 500 to 5000 times the concentrations of antimicrobial agents effective on their planktonic counterparts [1,2]. Biofilm formation is important for public health because this mode of growth is associated with the chronic nature of infections. Pathogenic bacterial colonization of dental implants and the infection of peri-implant tissues can lead to chronic bone destruction and may consequently lead to implant failure [3].

Peri-implantitis is an infectious disease of bacterial origin [4] that causes an inflammatory process in soft tissues around an osseointegrated dental implant in function and results in loss of the supporting bone [5]. The traditional treatment for peri-implantitis includes mechanical decontamination and local antiseptic or antibiotic treatment [6,7]. Suggested dental implant surface treatments are scaling, CO_2_ lasers, air abrasive powder, chlorhexidine or hydrogen peroxide irrigation, or local antibiotics. Nowadays, most of the information on the effectiveness of such interventions derives from case reports, so that no evidence-based consensus has been reached as to which option is clinically most advantageous.

Silver has the highest level of antimicrobial activity of all the heavy metals that have been recognized for thousands of years. The antimicrobial effect of silver has been demonstrated in numerous and varied applications against different types of microorganisms including bacteria, viruses, and protozoa. Gram-negative bacteria seem to be more sensitive to silver than Gram-positive species [8,9,10]. The bactericidal efficacy of silver ions is through the strong binding with disulfide and sulfhydryl groups found in the proteins of bacterial cell walls. Through these binding events, silver blocks the respiratory chain [10] then normal metabolic processes are disrupted, resulting in cell death.

Silver has been used in numerous medical applications [11]. Silver is effective against a number of oral bacteria including Gram-negative periodontal pathogens such as *Porphyromonas gingivalis*, *Prevotella intermedia*, and *Aggregatibacter actinomycetemcomitans* that cause inflammatory response of periodontal tissue [12]. The results of silver electrochemistry experiments have suggested that silver has potential as a chlorine alternative in drinking water disinfection in applications, in which chlorine may be considered too hazardous [13]. Silver has been used as an effective water disinfectant for several decades [14]. The World Health Organization (WHO) and the Environmental Protection Agency (EPA) consider silver as a non-toxic element to humans [15].

Biofilms are resistant to most antimicrobial agents used in clinical practice, which delays recovery from biofilm-associated infections in humans. Because of this failure of antimicrobial agents in biofilm-associated infection treatment, several researchers have investigated novel and innovative therapeutic approaches [16,17,18]. Recent approaches claim that electrical current can increase the efficacy of antibiotics and biocides [2,19,20]. It has been demonstrated that direct electric current substantially enhances the activity of antimicrobial agents in in vitro experiments (this has been defined as “bioelectric effect” [2]). The hypothetical mechanisms for the bioelectric effect include electric current disrupting the capacity of a biofilm for binding the antimicrobial agent and, thus, allowing penetration [19], electroporation increasing cell permeability [16,19], and electrolytically generated O_2_ enhancing biofilm metabolic activity [20]. The use of low electric currents with metal electrodes could inhibit bacterial growth in a liquid broth medium [21]. It was found that a silver anode would have the greatest inhibitory effect on bacterial multiplication and in fact, silver would continue to have this effect after current flow ceased. The silver ion was suggested to promote bactericidal effect. Electrically injected silver ions were shown to be at least as effective as an antibiotic. The evidence for the antimicrobial properties of silver ions with low electric currents was reported [22].

In an attempt to develop a process for the treatment of peri-implantitis, the effects of ionic silver and electrical treatment for surface disinfection and bacterial biofilm susceptibility on titanium implant surface was investigated in this work. An in vitro study was employed to represent the environment of dental implants. A trace concentration level of ionic silver combined with low-current electrical treatment to reduce the populations of *E. coli* biofilm-contaminated titanium surfaces was highlighted. The adverse effects on electrical polarized titanium surfaces were examined.

## 2. Results

### 2.1. Silver Susceptibility of Biofilm—Logarithmic Reduction Factor (LF)

**Figure 1:**
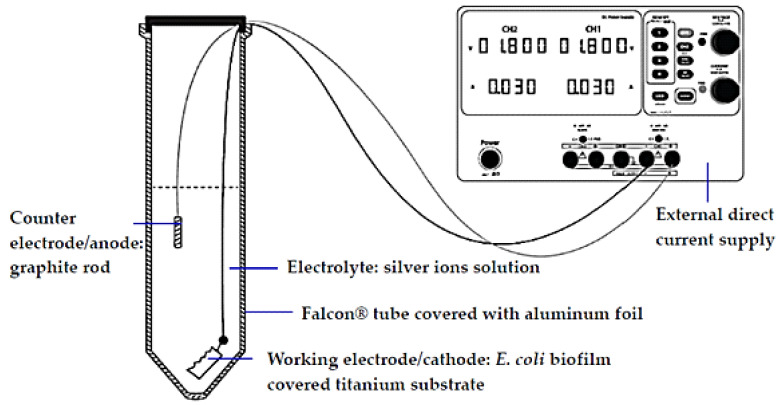
Schematic diagram of the electrochemical cell setup.

**Figure 2:**
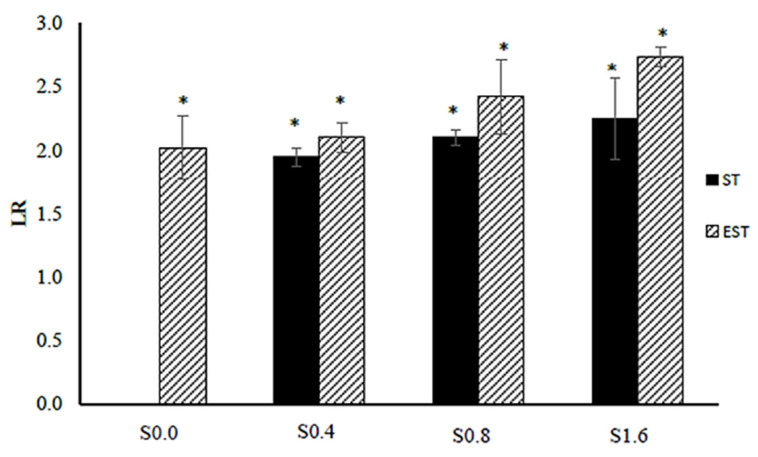
Logarithmic reduction (LR) of *E. coli* ATCC 25922 after the ionic silver treatments (ST-Sxx) and the electrical-enhanced silver treatments (EST-Sxx) in comparison to the non-ionic and non-electrical treatment (ST-S0.0). The values are expressed as mean ± SD_LR_ (*n* = 3); the symbol * indicates a statistically significant difference compared to ST-S0.0 at *p* < 0.05.

In this work, the surface disinfection and biofilm susceptibility were investigated by applying ionic silver of 0.4–1.6 µg/mL and cathodic voltage-controlled electrical treatment of 1.8 V and a current of 30 mA, for 60 min to *E. coli* ATCC 25922 biofilm on titanium substrate as a pathogenic Gram-negative bacteria model. Details of experimental treatments are shown in Table 1. The schematic diagram of the electrochemical cell setup is shown in Figure 1. The numbers of *E. coli* cells (in CFU/mL) after the silver treatments (ST-Sxx) and electrical-enhanced silver treatment (EST-Sxx) in comparison to the non-electrical and non-ionic silver treatment (ST-S0.0) are shown in Table 2. The effect of the treatments on *E. coli* biofilms was measured via the logarithmic reduction (LR) in the numbers of CFU/mL. The values of LR of *E. coli* viable cells after the silver treatments and electric-enhanced silver treatments at different ionic silver concentrations are shown in Figure 2. A significant reduction in cell viability was detected for all treatments at various silver concentrations. Results showed that the values of LR relatively increased as ionic silver concentration increased. The LR values after the ionic silver treatments (ST-Sxx) and the electric-enhanced silver treatments (EST-Sxx) ranged from 1.94–2.25 to 2.10–2.73, respectively. The electric-enhanced silver treatments showed higher LR values than those of the ionic silver treatments for all concentrations of silver ions. The results revealed that the electrical treatment effectively enhanced the silver susceptibility of *E. coli* biofilms.

### 2.2. Effects on Morphology of Biofilm Cells

**Figure 3:**
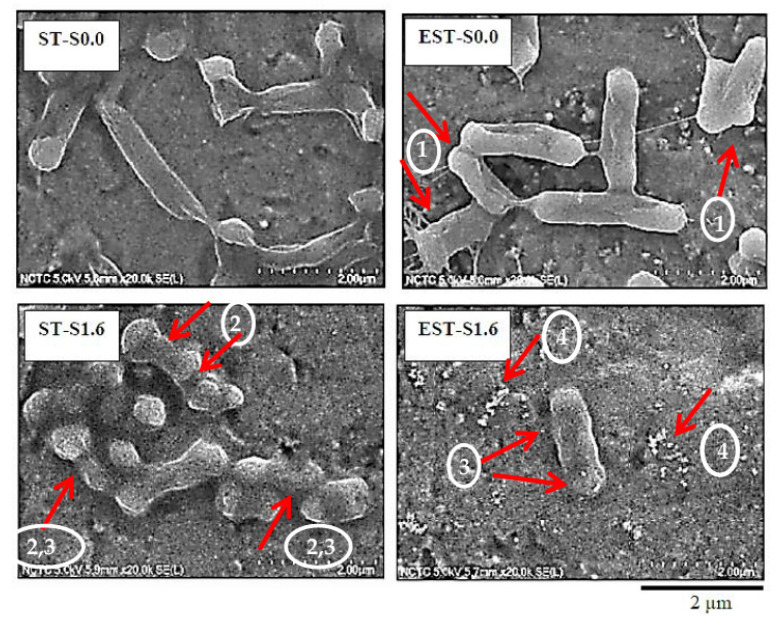
Morphology of *E. coli* ATCC 25922 cells of biofilm-contaminated titanium surface with non-ionic and non-electrical treatment (ST-S0.0) in comparison to those with electrical treatment (EST-S0.0), ionic silver 1.6 µg/mL without electrical treatment (ST-S1.6), and ionic silver 1.6 µg/mL with electrical treatment (EST-S1.6).

With the ionic silver and/or electrical treatment, a reduction in *E. coli* populations was observed. In Figure 3, the morphologies of *E. coli* biofilm cells after the treatments were characterized via FE-SEM. With non-electrical and non-ionic silver treatment (ST-S0.0), *E. coli* cells displayed a rod-like shape with smooth cell surfaces. The ionic silver treatment or/and the electrical polarization treatment induced morphological changes in *E. coli* cells. Arrow heads No.1 in Figure 3 show the bacterial cells with shorter length and significant structural deformation (arrow heads No.2). The damage of the outer membrane was subsequently observed (arrow heads No.3). The outer membrane disruption accompanied by blurring of the membrane wall boundaries (arrow heads No.3) and formation of cell debris (arrow heads No.4) were detected, especially under the treatment of ionic silver at 1.6 µg/mL with electrical treatment (EST-S1.6).

**Figure 4:**
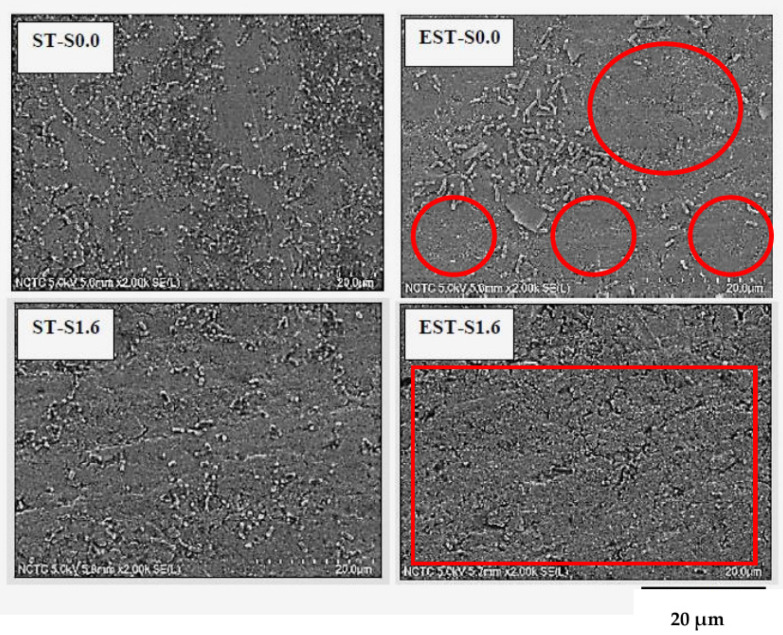
Morphology of biofilm-contaminated titanium surface under conditions with non-ionic and non-electrical treatment (ST-S0.0) in comparison to those with electrical treatment (EST-S0.0), ionic silver 1.6 µg/mL without electrical treatment (ST-S1.6), and ionic silver 1.6 µg/mL with electrical treatment (EST-S1.6).

The surfaces of *E. coli* biofilm cells treated with ionic silver at 1.6 µg/mL, with and without cathodically electrical polarization at 1.8 V and 30 mA for 60 min (ST-S1.6 and EST-S1.6, respectively) in comparison to those of the non-electrical and non-ionic silver treatment (ST-S0.0) and the electrical and non-ionic silver treatment (EST-S0.0) are shown in Figure 4. The change of biofilms on the titanium substrates with the treatments was noticed. As compared to ST-S0.0, ST-S1.6 showed lower number of *E. coli* cells; whereas, EST-S0.0 showed some clear surface areas (red circles) and some remaining biofilm cells. EST-S1.6 show almost entirely clear surface area (red rectangular area). After the treatments, *E. coli* populations on the titanium surface listed in order from low to high were as follows: EST-S1.6 < ST-S1.6 < EST-S0.0 < ST-S0.0. Therefore, the ionic silver treatment combined with electrical polarization could effectively help remove bacterial biofilms from the titanium surface.

### 2.3. Effect of Electrical Treatment on Titanium Surface

**Figure 5:**
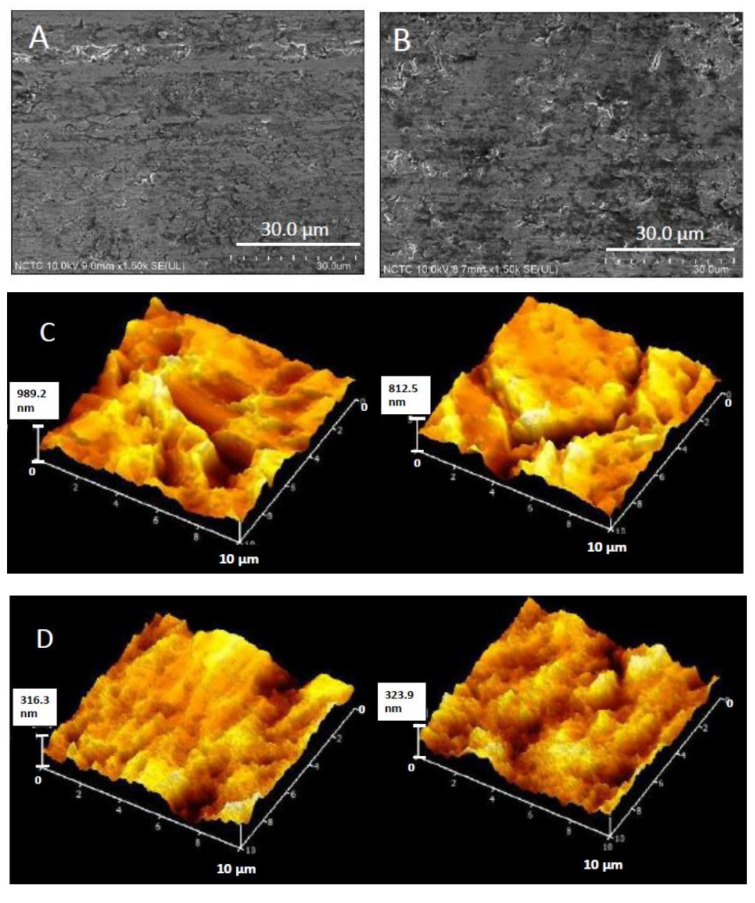
Surface morphology of titanium substrate before (**A**) and after (**B**) being treated with the cathodically electrical polarization; surficial roughness before (**C**) and after (**D**) being treated with the cathodically electrical polarization.

Effect of the cathodically electrical treatment on titanium substrate for 60 min on surface morphology and surficial roughness are shown in Figure 5. Overall, no considerable change on surface morphology was observed after the treatment (Figure 5A,B). However, as shown in Figure 5C,D, lower surface roughness values of the substrate after being treated with the cathodically electrical polarization were detected by atomic force microscopy. The average surficial roughness of the titanium substrate with the electrical treatments was 322 ± 23 nm, which was less than that of the non-electrical treatments (996 ± 180 nm). Therefore, the titanium surface was relatively smoother after the electrochemical treatment. 

### 2.4. Biocompatibility

**Figure 6:**
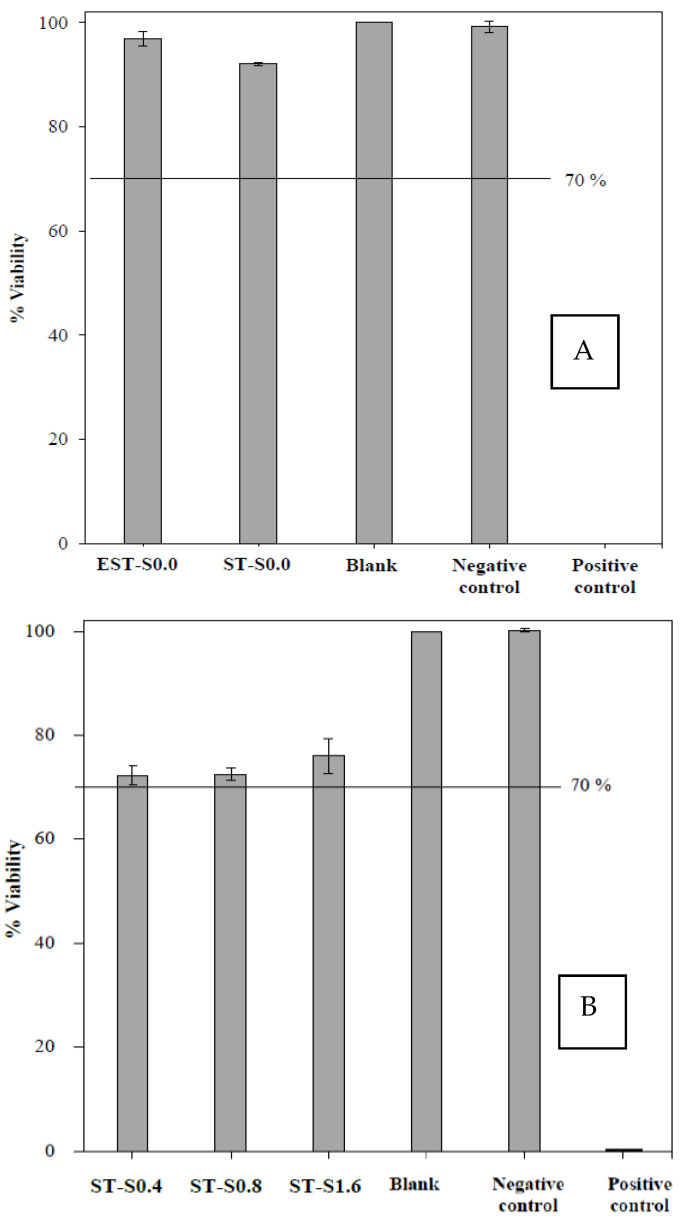
The biocompatibility on L929 mouse fibroblast cells: (**A**) the electrical treatment with non-ionic silver (EST-S0.0), the non-ionic and non-electrical treatment (ST-S0.0) in comparison to blank and controls; (**B**) the ionic silver treatments (ST-S0.4, ST-S0.8 and ST–S1.6) in comparison to blank and controls. The values are expressed as mean ± SD (*n* = 3).

Regarding biomaterial application, the MTT assay for biocompatibility test was performed. MTT assay, a quantitative test of ISO (ISO 10993-5: 2009), has been widely used to evaluate the biocompatibility of medical devices. The biocompatibility assay of titanium substrate under the electrical treatment with non-ionic silver (EST-S0.0) and the non-electrical with non-ionic silver treatment (ST-S0.0) in comparison to blank and controls was investigated on L929 mouse fibroblast as shown in Figure 6A. Under the treatments, in the case that the cell viability is higher than 70%, it is considered as non-cytotoxic. As shown, the viability of the cells with EST-S0.0 treatment was found to be 96%; thus, the cathodically electrical polarized titanium treatment in this study was considered non-toxic. The results for the biocompatibility test of the ionic silver treatments in the applied concentration range of 0.4 to 1.6 µg/mL on the viability of L929 mouse fibroblast cells are shown in Figure 6B. The results also suggested no cytotoxic effects of the applied ionic silver treatment.

### 2.5. pH Alteration of Titanium Substrate Polarization

**Figure 7:**
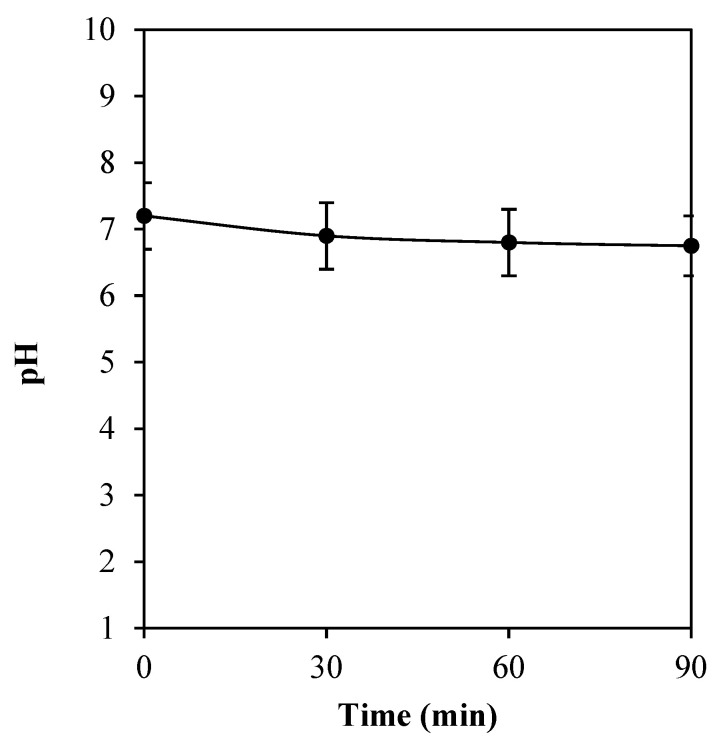
The pH alteration of cathodically electrical polarization of titanium substrate for 30, 60, and 90 min. The values are expressed as mean ± SD (*n* = 3).

In Figure 7, the pH alteration of cathodically electrical polarization of titanium substrate is displayed. A small decrease in pH value was found, as the operating time of the treatment increased. Initially, the pH value was at 7.2. Thereafter, at 30, 60, and 90 min of the operation, a slight decrease in pH to 6.9, 6.8, and 6.8, respectively, was observed. Herein, the pH of the system was found to be not considerably changed with the electrical treatment.

## 3. Discussion

From a clinical point of view, this surface disinfection technique using a low concentration of ionic silver (0.4, 0.8, and 1.6 µg/mL) that is lower than the toxic level to human mesenchymal stem cells (2.5 µg/mL) [23] and has a low potential (1.8 V) and currents (30.0 mA) of DC should be safe and appropriate for clinical application. The treatment by using ionic silver at extremely low concentration has many advantages, such as low toxicity, good biocompatibility with human cells, long-term antibacterial activity, and low bacterial resistance. Ionic silver exhibited effectively in inhibiting bacteria growth since it damaged the DNA of both Gram-positive and Gram-negative bacteria. It was reported that silver ions could cause bacterial inactivation in vitro by binding both to microbial DNA and to the sulfhydryl groups of the metabolic enzymes of the bacterial electron transport chain [24]. It is acknowledged that the free/unbound active ionic silver can enhance antimicrobial activities. It is highly reactive and can form a coordinated ligand with other ions in the solutions. For instance, when ionic silver was trapped with chloride ions (Cl^-^), silver chloride was released into the solution and dispelled the antimicrobial activity. In this present work, it is evident that ionic silvers in aqueous solution exhibited a proper and predictable antibacterial activity. Previously, the possibility of using a high concentration of ionic silver (≥25 µg/mL) for bacterial pathogen treatments has been reported [12,25].

The effect of the optimized electrochemical treatment was assessed on mammalian cells and tissues using histopathological evaluation of oral mucosa directly exposed to the current [26]. Under the consideration of electrical treatment, the currents below the hazardous current limit (<88 mA of DC) should be safe and tolerable for human beings. Microscopically, the tissues exposed to the optimized electrochemical treatments showed similar histological features to that of negative controls of nontreated tissues, confirming the safety of this protocol to cells and tissues [26]. Numerous in vitro reports have described the antimicrobial effects using direct electrical simulation [17,27,28,29,30,31]. Previously, it was reported that the application of cathodic voltage-controlled electrical stimulation (CV-CES) at 1.8 V (1 h) to titanium implants could reduce the MRSA CFU recovered from the bone and implant [31]. CV-CES at 1.8 V in combination with a 5-week course of vancomycin therapy could effectively decrease the implant and bone bacterial burden.

Based on electrochemistry principle regarding to clinical dental implant applications, the application of silver disinfection of *E. coli* biofilm-contaminated titanium surface was investigated in the present work. Generally, the biofilm-contaminated titanium implants as a working electrode and the graphite probe as a counter electrode are inserted into the peri-implant pocket. The ionic silver electrolyte/solution, which filled into the peri-implant pocket of infected bony defects around the implants, transfers the direct current to complete electrochemical reaction. It has been reported that human body has a poor conductivity which the resistance of 1–2 KΩ, whereas the titanium implants have higher conductivity in which there is resistance of 75–135 Ω [32,33]. The difference in current conductivity takes the possibility for clinical safe application. The current generated from the implants and the electrodes will pass through the ionic silver electrolyte/solution in the peri-implant pocket, the most conductive elements, and avoid the normal, healthy peri-implant tissue that are more resistant to electrical currents. The lower resistance between electrode and tissue interfaces is desired to reduce energy loss of the communication current [32]. In general, titanium as a working electrode has a lower resistance between the tissue–electrode interfaces. However, the resistance was found to be slightly increased due to the oxidation reaction of the titanium surface. Though, titanium is a promising electrode as a conductive medium for human body.

*E. coli* is a Gram-negative bacterium, which is a facultative anaerobic in nature and can be easily grown in a laboratory. It has been considered a model organism related to biological engineering and industrial microbiology [34,35]. *E. coli* biofilm is considered as a cause for indwelling medical device relating the infectivity. It is the major cause of many intestinal infections. Previously, the 6-day-age *E. coli* ATCC 25922 biofilm was employed as a Gram-negative anaerobe pathogenic biofilm to establish the titanium dental implant [36,37]. A biofilm is an aggregate of microorganisms that live together as a community and are often found attached to solid surfaces in moisture environment. The microbes in a biofilm secrete a variety of protective substances, which is called the extracellular polymeric substances (EPS) that enhance their survival efficiency. Biofilm renders difficulty in the penetration of conventional antibiotics; thus, the biofilm cells can be less susceptible to the antibiotics [35,38,39].

In this study, it was revealed that the electrical treatment in a low-intensity DC electric range (1.8 V and a current of 30 mA, for 60 min) could be applied for the treatment of *E. coli* biofilm-contaminated titanium surface. An application of direct low electrical current could be safely used in humans for fracture healing. Titanium is an extremely active element. Under the electrical treatment, the electrochemical (oxidation/reduction) reactions were induced and generated the oxidative species as follows [40].

Ti → Ti^2+^ + 2e^−^,
2TiO_2_ + 2e^−^ → Ti_2_O_3_ + O^2−^
Ti_2_O_3_ + 2e^−^ → 2TiO + O^2−^
TiO + 2e^−^ → Ti + O^2−^

At the anode, water is oxidized to oxygen gas and hydrogen ions (H^+^). At the cathode, water is reduced to hydrogen gas and hydroxide ions (OH^−^)

Oxidation reaction (anode): 2H_2_O → O_2_ + 4H^+^ + 4e^−^
Reduction reaction (cathode): 2H_2_O + 2e^−^+ → H_2_ + 2OH^−^

In this study, the synergistic effect between ionic silver and electrical stimulation to kill/remove biofilm cells was demonstrated. The values of LR using the synergistic effect were significantly higher than those of singly ionic silver treatment. When the conventional silver treatment was applied separately, the values of LR were relatively lower than those combined with the electrical treatment. The mechanism of action to kill/remove biofilm cells of silver ions in conjunction with the electric field should be due to the increase in cell membrane permeability caused by the externally applied electrical field that might enhance the penetration of silver ions through biofilms. The electrostatic force from a negatively polarized surface could also promote detachment of attached bacteria or biofilm [41,42]. In addition, the electrochemical formation of hydrogen gas bubbles at the porous biofilm/substrate interface might increase the biofilm detachment [43]. Previously, within a low-intensity DC electric field, the efficacy of biocides and antibiotics in killing bacterial biofilm can be radically enhanced [19,44]. This is due to an increased fluidity of the matrix allowing a better penetration of the antibiotic. Similar works have been conducted via an electrical enhancement of *E. coli* biofilm. Electrical enhancement of *E. coli* biofilm killing by gentamicin or oxytetracycline was reported [45]. Furthermore, the results of in vitro biocompatibility test on L929 mouse fibroblasts confirmed that the cathodically electrical polarized titanium treatment under a low potential (1.8 V) and currents (30.0 mA) of DC in this study was non-toxic. The evaluation of biocompatibility of the ionic silver treatments at very low concentration (0.4 to 1.6 µg/mL) also suggested no significant cytotoxic effect of the applied ionic silver treatment.

The voltage-dependency observed for pH is related to the voltage-dependent reduction in sustained bacteria attachment [46]. The relationship between bacterial CFU and the microenvironment pH following application of electrical stimulation have been reported previously [47,48]. At anode, the production of H^+^ resulting in a higher acidity of the surrounding was obtained; whereas, at cathode, the production of OH^−^ was found to induce a higher basicity in the surrounding. In this study, cathodic voltage-controlled electrical stimulation (CV-CES) at 1.8 V was applied for 60 min at the titanium substrate, which functioned as a cathode. It was found that the pH of the solution had slightly changed. The effect of the electrical treatment on a slight decrease in pH of electrolyte should be due to the increase in [H^+^] in the electrolyte. However, it was previously recorded as a significant increase in the pH using CV-CES of titanium for prevention of methicillin-resistant *Staphylococcus aureus* and *Acinetobacter baumannii* biofilm infections at −1.8 V for 2, 4, and 8 h of operation time [46]. A decrease in pH could also be due to the adherent bacteria and their metabolic activity. In addition, differences in the applied method and operating conditions could provide different results. Additionally, the slight change in surface roughness of titanium substrate after being treated with the cathodically electrical polarization was detected by atomic force microscopy, which might result from the reduction reaction at titanium oxide layer on the surface of titanium substrate.

## 4. Materials and Methods

### 4.1. Chemicals and Electrolytes

The volumetric solution of silver nitrate, 0.1 mol/L (0.1 N), supplied by PanReac AppliChem^®^ was used to prepare silver aqueous solution in the concentration range of 0.4, 0.8, and 1.6 µg/mL. The normal saline solution (0.9%) (Klean&Kare^®^) was used as none silver solution.

### 4.2. Substrate Preparation

Square titanium substrates (10 mm × 10 mm and thickness of 1 mm) were laser cut from a medical quality titanium alloy Ti6Al4V sheet (ASTM Grade 5). The customized titanium substrates were fabricated via the electric conductor loop formed by stainless steel wire (0.1 mm in diameter) at an angle of each square titanium substrate. After that, the substrates were sonicated in deionized water (DI) and degreased in acetone and ethanol, then sterilized by autoclaving at 121 °C for 30 min.

### 4.3. Biofilm-Contaminated Titanium Substrate

*E. coli* ATCC 25922 was used as pathogenic Gram-negative bacteria model. *E. coli* biofilms were formed on customized titanium substrates according to previous study [36,37]. Procedures carried out can be briefly described as follows. Firstly, small aliquots of bacteria (test cultures stored at −80 °C) were cultured in a 50 mL Falcon^®^ tube which contained 20 mL of a growth medium lysogeny broth (LB). Then, the solution was incubated at 37 °C and 100 rpm, for 16 h, until the exponential growth phase was reached. The values of OD_600_ in the range of 0.5–1.5 was checked every 4 h. Subsequently, these bacterial suspensions were grafted directly on titanium samples as a biofilm. The prepared titanium substrates were placed in a 50 mL Falcon^®^ tube which contained the solutions of LB medium (25 mL) and overnight cultures (2.5 mL) (OD_600_ 0.5). The titanium substrates in culture suspension were incubated at 37 °C for 21 h. After that, the titanium substrates were positioned in another 50 mL Falcon^®^ tubes in 25 mL of LB medium and incubated at the same conditions. This procedure was repeated five times.

### 4.4. Electrical Polarization Set-Up and Cell Voltage

The experimental setup of the custom-built electrochemistry cell is depicted as shown in Figure 1. A 50 mL of Falcon^®^ tube covered with aluminum foil for dark experimental condition was used as a custom-built electrochemistry cell container for titanium substrate polarization. The experimental were conducted in the cell container. *E. coli* ATCC 25922 biofilm-contaminated titanium substrates was used as cathode. A graphite rod (2 mm in diameter and 20 mm length) was used as anode. The cathodic voltage-controlled electrical was controlled using a laboratory power supply (GW Instek^®^, GPD-2303S series). The circuit inputs were checked with a digital high precision multimeter (Fluke 87 True RMS), every 20 min during polarization. The standard condition was applied as follows: 1.8 V and a current of 30 mA, for 60 min, over a junction between the titanium cathode and a graphite anode. The following parameters were investigated:

(i) Ionic silver treatment (non-electrical polarization) in aqueous solution. Herein, the concentration of ionic silver was varied at 0.0, 0.4, 0.8, and 1.6 µg/mL.
(ii) Ionic silver combined with electrical treatment in aqueous solution. The concentration of ionic silver was varied at 0.0, 0.4, 0.8, and 1.6 µg/mL.

### 4.5. CFU Assay (Viable Cell Quantification)

A colony forming unit (CFU) assay can be measured via following steps. Firstly, the silver-treated *E. coli* ATCC 25922 biofilms were extracted from the 50 mL Falcon^®^ tube by washing in ultra-pure water and placed in a vortex machine for 5 min, to release the *E. coli* biofilm cells from the surface. Then, the CFU were enumerated by plating a 10 µL of serial 10-fold dilutions onto the LB agar plates. Then, the plates were incubated at 37 °C, for 18 h, after that, the CFU were counted.

The density of live bacteria (CFU/mL) on each plate was determined. Each experiment was repeated three times and the results were averaged. The biofilm reduction was determined using the logarithmic reduction (LR) as shown in Equation (1) [49,50]. The values are expressed as mean ± SD_LR_ (*n* = 3).

LR = (mean of log [CFU/mL] for untreated samples) − (mean of log [CFU/mL] for treated samples)(1)

SD_LR_ = [(SD^2^_un_/n_un_) + (SD^2^_tr_/n_tr_)]^1/2^ (2)

### 4.6. Biofilm-Contaminated Surface Morphology

In order to examine the morphology of biofilm cells and titanium surfaces before and after all treatments, the titanium coupons were scanned before and after the treatments using SEM (FE-SEM SU5000, Hitachi, Japan). The silver-treated biofilm-contaminated were aseptically extracted from the stimulating culture tube, after 60 min of all treatment processes. The sample preparation for SEM characterization is described as follows. The silver-treated biofilm-contaminated coupons were fixed overnight with 2.5% glutaraldehyde and 2% paraformaldehyde in 0.1 M phosphate buffer. Then, 0.1 M of phosphate buffer was rinsed (3 × 10 min each). Thereafter, the coupons were dehydrated gradually by being washed sequentially with 50, 75, 85, and 95% ethanol solution (10 min each) and 100% ethanol solution (3 × 10 min each). Hexamethyldisilizane (HMDS) was used for overnight drying. Finally, samples were then sputter-coated with gold prior to FE-SEM characterization [49,51].

### 4.7. Surface Characterization of Electrically Polarized Titanium Surface

Samples for SEM and Atomic Force Microscopy (AFM) were immersed in 25% glutaraldehyde overnight, afterwards dipped ten times in 50% EtOH [50,52] and dried in a vacuum desiccator for at least 2 h. Morphology of the samples was studied using a scanning electron microscope (FE-SEM SU5000, Hitachi, Japan), at an acceleration voltage of 10–15 kV. Samples were sputtered with ~10 nm of gold. Surface topography was acquired on an atomic force microscope (AFM5500M, Hitachi, Japan) in semi-contact mode with a silicon tip and at different scanning areas at a scan rate of 1 Hz. The experiments were conducted in air and at room temperature.

### 4.8. Biocompatibility

L929 mouse fibroblast cells and minimum essential medium (MEM, Sigma-Aldrich), were employed to study the conventional cell culture assay. Based on the MTT assay technique, the cytotoxicity assay was quantitatively performed. According to ISO standard (ISO 10993-5: 2009(E)), polyurethane film containing 0.1% zinc diethyldithiocarbamate (ZDEC): RM-A and Thermanox (Nunc) coverslip were used as a positive and negative control material, respectively. The viability of extract-treated cells <70% of the blank, indicated that the sample has a cytotoxic potential. Thus, the cell viability must be equal to or greater than 70% regarding a non-cytotoxic consideration.

### 4.9. pH Measurements

The pH alteration of the electrical conducting media (0.9% normal saline solution, Klean&Kare^®^) in the electrical polarization of none *E. coli* biofilm-contaminated titanium substrate was measured at 30, 60, and 90 min of the operation.

## 5. Conclusions

Herein, the synergistic effect of ionic silver and cathodic voltage-controlled electrical stimulation was investigated. The method is based on the use of low electrical potential (1.8 V, 30 mA) and very low concentration of ionic silver (0.4–1.6 µg/mL), which is considered safe for humans and sufficient to disinfect *E. coli* biofilm. The treatments for 60 min to *E. coli* biofilm-contaminated titanium surface significantly reduced the viability of *E. coli* cells. The synergistic effect of combining ionic silver and electrical treatment could promote the efficient disinfection of *E. coli* biofilm-contaminated titanium surface. The LR values after the electric-enhanced silver treatments for 60 min were in the range of 2.10–2.73. No considerable change in the surface of titanium substrates and only a slight decrease in pH were observed during the electrical polarization. The treatments showed no cytotoxic effects on L929 cells by MTT assay.

## Figures and Tables

**Table 1:** Details of experimental treatments.

Experimental Group	Treatment	Symbol
Silver treatment	Ionic silver xx µg/mL in aqueous solution for 60 min	ST-Sxx ^1^
Electrical-enhanced silver treatment	Ionic silver xx µg/mL in aqueous solution combined with electrical treatment ^2^ for 60 min	EST ^2^-Sxx ^1^

^1^ Concentration of silver ions (xx) is varied: 0.0, 0.4, 0.8, and 1.6 µg/mL. ST-S0.0 is the non-electrical and non-ionic silver treatment; EST-S0.0 is the electrical and non-ionic silver treatment. ^2^ Electrical treatment for cathodically electrical polarization is performed at 1.8 V and 30 mA for 60 min.

**Table 2:** Numbers of viable cells (CFU/mL) of *E. coli* ATCC 25922 biofilms after the ionic silver treatments (ST-Sxx) and the electrical-enhanced silver treatment (EST-Sxx) with different ionic silver concentration (0.0, 0.4, 0.8, and 1.6 µg/mL) in comparison to the non-ionic and non-electrical treatment (ST-S0.0). The values are expressed as mean ± SD (*n* = 3).

CFU/mL	S0.0	S0.4	S0.8	S1.6
ST	1.14 × 10^8^ ± 1.20 × 10^7^	1.33 × 10^6^ ± 3.21 × 10^5^	9.20 × 10^5^ ± 1.77 × 10^5^	1.09 × 10^6^ ± 7.15 × 10^5^
EST	1.55 × 10^6^ ± 9.31 × 10^5^	9.77 × 10^5^ ± 3.53 × 10^5^	7.67 × 10^5^ ± 7.04 × 10^5^	2.18 × 10^5^ ± 5.98 × 10^4^

## Data Availability

Not applicable.
